# Aging and DNA damage in humans: a meta-analysis study

**DOI:** 10.18632/aging.100667

**Published:** 2014-06-05

**Authors:** Jorge Pinto Soares, António Cortinhas, Teresa Bento, José Carlos Leitão, Andrew R. Collins, Isabel Gaivã, Maria Paula Mota

**Affiliations:** ^1^ CIDESD, University of Trás-os-Montes and Alto Douro, Vila Real, Portugal; ^2^ University of Trás-os-Montes and Alto Douro, Vila Real, Portugal; ^3^ Department of Nutrition, Faculty of Medicine, University of Oslo, Oslo, Norway; ^4^ CECAV - Genetic and Biotechnology Department, University of Trás-os-Montes and Alto Douro, Vila Real, Portugal

**Keywords:** DNA damage, age, humans, meta-analysis, lifestyle

## Abstract

Age-related DNA damage is regarded as one of the possible explanations of aging. Although a generalized idea about the accumulation of DNA damage with age exists, results found in the literature are inconsistent. To better understand the question of age-related DNA damage in humans and to identify possible moderator variables, a meta-analysis was conducted.

Electronic databases and bibliographies for studies published since 2004 were searched. Summary odds ratios (ORs) and 95% confidence intervals (CIs) for age-related DNA damage were calculated in a random-effects model.

A total of 76 correlations from 36 studies with 4676 participants were included. Based on our analysis, a correlation between age and DNA damage was found (r = 0.230, p = 0.000; 95% confidence interval = 0.111 - 0.342). The test for heterogeneity of variance indicates that the study´s results are significantly high (Q (75) = 1754.831, p = 0.000). Moderator variables such as smoking habits, technique used, and the tissue/sample analyzed, are shown to influence age-related DNA damage (p=0.026; p=0.000; p=0.000, respectively). Nevertheless, sex did not show any influence on this relation (p=0.114).

In conclusion, this meta-analysis showed an association between age and DNA damage in humans. It was also found that smoking habits, the technique used, and tissue/sample analyzed, are important moderator variables in age-related DNA damage.

## INTRODUCTION

Aging has been defined as a progressive organic functional decline, with loss of homeostasis and increasing probability of illness and death [1]. Although applied research on aging had resulted in considerable scientific knowledge with regard to the related causes, it is still subject to numerous debates and contradictions. Indeed, in most cases, it is difficult to understand how a particular variable is a possible cause or consequence of aging. Some studies have suggested an age-related accumulation of macromolecular damage, which may cause progressive and irreversible physiological attrition and homeostasis loss, accelerating aging. In addition, considering the important role of DNA in living organisms and regarding the changes that occur in this macromolecule throughout life, the question arises, whether DNA modifications should be considered a central factor of aging. Moreover, it is not clear if these damages are a possible cause or an expression of aging. Since the first study of Failla [2], numerous other investigations have been performed and different assays to measure DNA changes have been developed. These assays allow the assessment of some age-related DNA changes, as well as other associated variables that could interfere with DNA damage accumulation.

Many theories of aging are based on DNA changes, including the Intrinsic Mutagenesis Theory, Somatic Mutations Theory and DNA Repair Theory [3, 4]. Other theories also explain the age-related changes in DNA as a consequence of stochastic events. The Oxidative Stress Theory is, perhaps, the main stochastic explanation of DNA and other macromolecular damage accumulating with age [4]. Despite the importance of the age-related DNA damage accumulation, some researchers argue that aging is not caused by the accumulation of damage but is the result of continued activity (cell hyperfunction) of pathways and processes during adulthood that evolved to optimize development to this life stage [5, 6]. According this theory, the same pathway, which drives developmental growth, later drives aging and associated diseases [7]. Cell hyperfunction is driven by the nutrient-sensitive signaling network that controls growth (and thereby, reproduction), and includes the insulin, insulin-like growth factor 1 (IGF-1), and in particular, the target of rapamycin (TOR) kinase pathways [6-8]. Thus, accumulation of DNA damage is a consequence of aging and not a leading cause.

Although DNA is not the only target changed with aging, taking account of the major role of this macromolecule in the regulation of all cellular structures and its own cell cycle, DNA damage has been studied with particular attention. The alterations could have several consequences for genome stability with repercussions on cellular component synthesis, cell cycle machinery and signaling pathways that control cell cycle arrest, and programed cell death or apoptosis [9]. The consequences of DNA damage will depend on the type of damage, genes affected and type of cell and tissue damaged.

The prevailing view is that there is a tendency for an age-related DNA damage accumulation. However, on examination, results of studies show inconsistency [10-13]; it is possible that confounding factors influence this relation and explain some of the inconsistency.

Considering the complexity of aging, it must be emphasized that aging does not happen in the same way in different individuals, nor in the same way in all cell types and tissues of the same individual. Moreover, aging is a life-long process, influenced continually by environmental conditions. Factors such as diet, lifestyle, exposure to radiation and genotoxic chemicals seem to have a significant influence on the relationship between cumulative DNA damage and age [14-16].

Methodological factors might have also influenced the observed results [14-16]. Indeed, different assays may be used to measure DNA damage. Furthermore, the measured DNA damage could reflect changes in the causative factors, and/or changes in DNA protection and/or changes in DNA repair capacity. It must also be noted that the type of cell and tissue used could reflect different aging rates within the organism.

Although there are several excellent narrative reviews on age-related nuclear DNA damage [17-20], they usually refer to individual animal and humans studies and, as far as we know, no meta-analytic technique has been used to estimate the extent of effect of potential moderators on age-related DNA damage in humans. Thus, the overall goal of this paper is to address this important gap in the literature. The first aim of this review is to provide a summary of age-related changes in nuclear DNA in humans. The second aim is to examine the effects of some moderators associated with DNA damage. The third aim is to discuss promising directions for future researches in the light of our findings.

## RESULTS

An initial search using the keywords described located 2953 studies. After reading titles and abstracts, the number of studies was reduced to 267. In the final refinement of the research, applying inclusion and exclusion criteria, only 36 studies fulfilled all necessary requirements.

### Study Analysis

**Figure 1:**
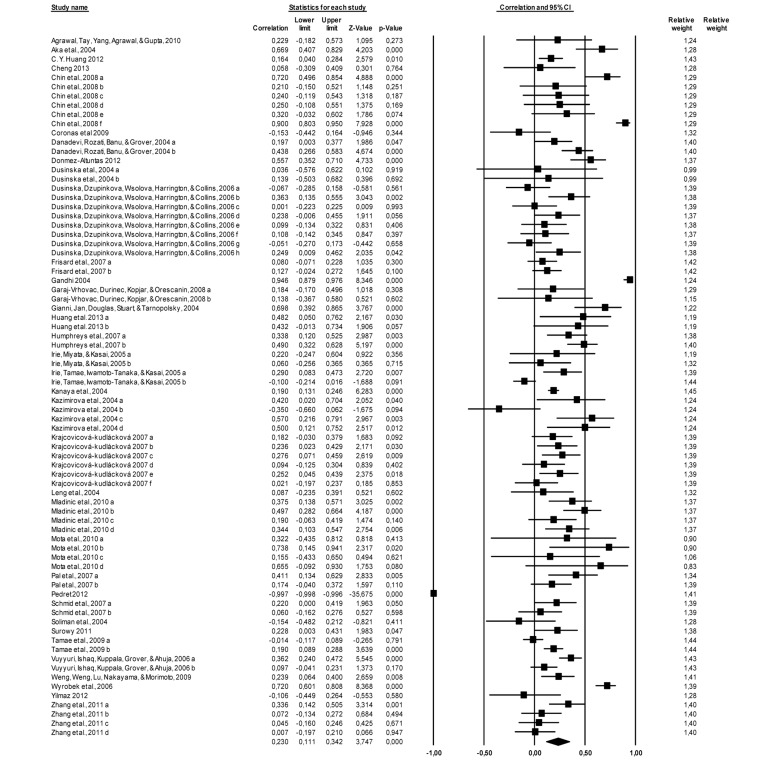
Forest plot, the effect size (r) of each study (relative weight of each study in the age-related DNA damage). IC=confidence interval. a, b, c, d, e, f, g, h – different measured endpoints from the same study [10-15, 21-48].

The results indicated a significant and positive association between age and DNA damage in 76 correlations of the 36 studies (N = 4676) r = 0.230, p = 0.000 (95% confidence interval = 0.111; 0.342). A test for heterogeneity of variance indicates that the results of the study are significantly higher than would be expected, Q (75) = 1754.831, p = 0.000. The effect size of each study can be seen in Figure 1.

The analysis of the moderator variables is shown in Table 1. As can be seen, tobacco use, sample/tissue and technique, but not sex, are identified as moderator variables.

**Table 1:** Effect size of the moderator variables (Sex, Tobacco, Sample/Tissue, and Technique) on age-related DNA damage

Moderator	Value	K	R	95%CI	*P*	*p* between groups
Sex	Female	25	0.116	0.05; 0.18	0.000	0.114
	Male	14	0.177	0.13; 0.22	0.000	
Tobacco	Non-Smokers	26	−0.043	−0.10; −0.02	0.162	0.026
	Smokers	2	0.176	−0.01; 0.35	0.058	
Sample/Tissue	Buccal	4	0.229	0.14; 0.32	0.000	0.000
	Mammary	1	−0.106	−0.45; 0.26	0.580	
	PBMC	57	0.235	0.20; 0.27	0.000	
	Spermatozoa	3	0.392	0.28; 0.49	0.000	
	Urine	11	−0.007	−0.05; 0.03	0.711	
Technique	SCSA	1	0.720	0.60; 0.81	0.000	0.000
	SCGE–CA	47	0.208	0.17; 0.24	0.000	
	ELISA	7	−0.083	−0.14; −0.03	0.002	
	HPLC	8	0.072	0.02; 0.12	0.007	
	MN	11	0.268	0.19; 0.34	0.000	
	SCE	2	0.831	0.73; 0.90	0.000	

PBMC – peripheral blood mono-nuclear cells; SCSA – sperm chromatin structure assay; SCGE–CA – Single cell gel electrophoresis- Comet Assay; ELISA - enzyme-linked immunosorbent assay; HPLC – High Performance/Pressure Liquid Chromatography; MN- micronucleus; SCE – sister chromatid exchange.

## DISCUSSION

DNA changes associated with age have been claimed as one of the main possible causes of aging. These alterations may result in genetic instability, mutagenesis, disease, and cell death. Despite its popularity, this association has a lack of consensus in the literature apparently due to several factors such as sample characteristics, technique and methods used. To clarify this relation, we conducted a meta-analysis to investigate the association between age and DNA damage in humans. Our main finding is a positive association between age and DNA damage in humans, both in males and females. So, aging in humans is accompanied by an increase in general DNA damage.

However, the association found is weak and as the Cochran's Q statistic and the I^2^ statistic revealed a high heterogeneity between studies. To better understand this association, there are some points that must be taken into consideration. First of all, this weak association implies that there are other variables which may influence age-related DNA damage. Variables suggested in the literature include sex, smoking, alcohol consumption, physical exercise, nutrition, psychological stress, etc. Secondly, the sample's characteristics, technique used, tissue and the type of damage analyzed could influence results. Considering this, some variables were analyzed here as possible moderator variables influencing age-related DNA damage. Several works have studied the effect of sex on DNA damage. Despite the fact that some studies have identified differences between sexes in DNA damage [23, 26, 36], our results based on analysis of 36 studies have shown no such differences. This means that both sexes show increased DNA damage with age, even though the absolute values could be different. In short, sex as a moderator variable has no influence on age-related DNA damage.

Although in the literature several lifestyle variables have been related with DNA damage, according to the results from the studies analyzed here, only smoking habit could be considered as a moderator variable; at the time of our analysis, there were not enough studies concerning the remaining variables. Since tobacco smoke contains known carcinogens, it seems plausible that smokers could accumulate more DNA damage with age, compared with non-smokers. Our analysis confirms this hypothesis, showing that smokers demonstrate more age-related DNA damage. Our results clearly suggest that smoking should be considered as a moderator variable in the age-related DNA damage studies.

As mentioned before, it is well established that aging does not occur at the same rate in the different organs. Accordingly, it might be expected that different sample tissues might show different age-related DNA damage. Considering the sample tissue studied, we found significant differences between them. Unexpectedly, mammary cells and urine samples have shown no there was only one study of mammary tissue cells and the urine sample results show high heterogeneity remaining cell types studied (buccal cells, peripheral blood cells and spermatozoa), less heterogeneity between studies was seen, and a positive correlation with age-related DNA damage was found.

Our results have shown that technique is a moderator variable when age-related DNA damage is studied, so that depending on the technique used, we might expect different results. Studies using ELISA have shown a weak negative association between age and DNA damage; HPLC, SCGE - CA and MN have shown a positive association but also weak; and a strong association for SCSA and SCE has been found, even though these last two were based on only one and two studies, respectively. Regarding these techniques, it is important to mention that ELISA and HPLC are the only two techniques which are used in urine and/or in tissues cells. This is of major importance because results outcomes are clearly different, since in the case of urine samples DNA damage is not analyzed directly, but rather the result of that damage in the overall body system. So our results illustrate the importance of careful interpretation, especially in comparisons of results from different studies.

There are some questions underlying age-related accumulation of DNA damage which must be taken into account. Firstly, it was our proposal to study the set of variables that might be associated with the DNA damage and aging. Though we were only able to consider smoking habits, other lifestyle variables should be evaluated in studies with human samples. Also, the methodology is important for understanding the results and interpreting heterogeneity. Sample characteristics as well as inclusion criteria are relevant to understand the results achieved. The range of ages included in a study is likely to influence the results – a wide range likely leading to more pronounced effects: in this meta-analysis age range was not considered due the variability of the studies designs and to the lack of information presented in the articles. Further, the weak association between DNA damage and age found in this meta-analysis raises the question of if accumulation of damage is determinative for the aging process. As mentioned above, it is not clear if age-associated damage is a cause or an expression of aging. The molecular damage theory has postulated that aging is caused by the progressive accumulation of damage; however according to the hyperfunction theory this damage is a consequence of aging and [7] thus does not necessarily limit lifespan [5, 7, 49]. Instead, the observed increased levels of damage are important for some pathologies, such as cancer, and are the result of hyperfunction. The hyperfunction theory even suggests that repair of molecular damage is important for increased longevity, but the involvement of any process for viability does not imply its role in aging [7]. In summary, there are emerging explanations concerning our understanding of aging, which provide a novel perspective on aging and the DNA accumulation of damage.

In conclusion, independent of the perspective theory of aging, our meta-analysis results show an age-related increase in DNA damage in humans. Furthermore, smoking habits, the sample/tissue and the analysis technique used are important moderator variables. There is a set of lifestyle variables which should be more carefully studied in longitudinal studies, since it seems that age-related DNA damage only explains a small part of aging. Future studies may also rely on the relevance of the age-related DNA damage and its possible role as a marker of biological aging.

## METHODS

We have conducted a meta-analysis based on the criteria described for meta-analyses and systematic reviews by Moher et al. [50].

### Data Source and search strategy

In order to achieve the largest number of publications, MEDLINE PubMed and Web of science (Web of ScienceSM; current Contents Connect) databases were used with the combination of the English key terms: “DNA”, “damage” and “age”, and all eligible studies between 2004 and “March 2013” were selected.

In addition, the reference lists in these articles were searched manually to find other relevant publications.

### Selection Criteria and Identification of Studies

The following inclusion criteria were used to select the articles to our study: articles in English, studies on nuclear DNA, male and/or female human studies, healthy subjects or studies with healthy control subjects, papers clearly describing the sources of cases and controls, and information given on the size of the sample and statistical values.

The exclusion criteria were: post-mortem studies, in vitro studies, studies of newborns, children and puberty, studies in exposed subjects without control group, and studies in non-healthy subjects without healthy control group.

### Data extraction

The following data were collected from each study: first author's surname; year of publication; cells and tissues analyzed; evaluation technique used; total number of cases and controls; age groups, DNA damage data.

### Selection of moderator variables

Moderator variables were largely based on the models presented by Cook-Cottone et al. (2009) [51].

Two authors of the present study were responsible for separately encoding each of the moderator variables, which were then compared to ascertain the percentage of agreement. The description of the criteria for coding is presented in the following section.

Many biomonitoring studies have shown that some indicators of genetic damage in different cells depend on various internal factors (such as sex and age) and external factors (such as smoke).

### Sex

Sex seems to be an important factor to be taken into account when conducting epidemiological studies. There is a lack of consensus on its influence on DNA damage. Adult women were reported to have lower levels of damage than men but others studies contradict this trend. In addition, it is not known whether sex might influence the age-related DNA damage.

### Smoking

It is well-known that some lifestyle behaviors have an influence on the stability and integrity of the DNA. In particular, smoking is a source of carcinogens which could have a significant effect on DNA damage. Despite that, the relationship between smoking and DNA damage as measured with the comet assay in PBMN cells is still inconsistent in the literature.

### Sample/Tissue

Aging does not occur in all biological structures in the same way. Indeed, some conflicting results could be explained by the different sample/tissue studied.

### Technique

Some of the inconsistency of results in the literature can be attributed to the different techniques used in different laboratories; these might vary in sensitivity, or might emphasize different kinds of lesion (for instance, SBs or oxidized bases).

### Statistical analyses

This meta-analysis included 36 studies. The strength of the association between DNA damage and age was assessed by the correlation values (r) described in the studies. In comparative studies the *p* values or *t tests* were analyzed to estimate the r value. In other works the correlation coefficient was calculated from mean and standard deviation values.

Analyses of results were performed using subgroups based on the moderator variables described above.

The statistical analyses were performed with the software Comprehensive Meta-analysis (CMA, version 2.2.048) [52]. Both the Cochran's Q statistic and the I^2^ statistic, to test for heterogeneity and to quantify the proportion of the total variation due to heterogeneity, were calculated. We chose the random-effects model due the great variability of the samples and techniques used.

Several methods were used to assess the potential publication bias. Visual inspection of funnel plot asymmetry was conducted. The Begg's rank correlation method [53] and the Egger's weighted regression method [54] were used to statistically assess publication bias (P<0.05 was considered statistically significant). The funnel plot shows a slightly asymmetrical distribution of points; however the rank-correlation test of Begg's (p=0.095) and Egger's (p=0.112) showed a non-significant publication bias.
